# Current concepts in genetics of nonsyndromic clefts

**DOI:** 10.4103/0970-0358.53004

**Published:** 2009

**Authors:** Jyotsna Murthy, L. V. K. S. Bhaskar

**Affiliations:** Department of Plastic Surgery, Sri Ramachandra Medical College, Chennai, India; 1 Department of Biomedical Sciences, Sri Ramachandra Medical College, Chennai, India

**Keywords:** Cleft, Genetics, Nonsyndromic clefts

## Abstract

Nonsyndromic cleft lip and palate is a complex genetic disorder with variable phenotype, largely attributed to the interactions of the environment and multiple genes, each potentially having certain effects. Numerous genes have been reported in studies demonstrating associations and/or linkage of the cleft lip and palate phenotypes to alleles of microsatellite markers and single nucleotide polymorphisms within specific genes that regulate transcription factors, growth factors, cell signalling and detoxification metabolisms. Although the studies reporting these observations are compelling, most of them lack statistical power. This review compiles the evidence that supports linkage and associations to the various genetic loci and candidate genes. Whereas significant progress has been made in the field of cleft lip and palate genetics in the past decade, the role of the genes and genetic variations within the numerous candidate genes that have been found to associate with the expression of the orofacial cleft phenotype remain to be determined.

## INTRODUCTION

Cleft lip and cleft palate are birth defects that affect the upper lip and roof of the mouth. It is the most common disease affecting children with variable phenotype. Some babies have only a cleft lip and many babies with cleft lip have a cleft palate as well. Cleft palate also can occur by itself without cleft lip (isolated cleft palate). Cleft lip/palate (CL/P) and isolated cleft palate are considered separate birth defects. Approximately 70% of CL/P cases are nonsyndromic occurring as an isolated condition unassociated with any recognizable anomalies, while the remaining 30% are of syndromic cases present in association with deficits or structural abnormalities occurring outside the region of the cleft.[1] Nonsyndromic CL/P is a common craniofacial malformation with a complex genetic component. From the available literature it can be seen that the incidence of orofacial clefting varies depending on geographical location, ethnicity, sex and even socioeconomic status. The incidence of CL and CLP is higher than CP. Incidence also varies markedly in different ethnicities. Though methods of ascertaining and classification criteria can influence incidence rates, it reported that North American Indians and Asians have the highest prevalence rates (sometimes as high as 1 in 500), while Caucasian populations have intermediate rates (1 in 1000) and African derived populations have the lowest prevalence rates (1 in 2500). Among the Asians the Japanese are believed to have the highest rates. The prevalence in Latino Americans is lower than that in Caucasians and Native Americans, yet it is still higher than African Americans. The frequency of CLP/ CP differs with regard to sex and side of clefting with 2:1 (male:female) ratio and a 2:1 (left side:right side) ratio for clefting in unilateral clefts. Cleft palate alone (CP) results in a prevalence rate of 5.5 to 6.6 per 10,000 births approximately and has a 0.73:1 (male: female) ratio among the Caucasian population.

Many sub-phenotyping features related with nonsyndromic cleft lip and palate have been studied, such as handedness, dental anomalies and dermoglyphic features. To date several studies have reported higher incidence of non-right-handedness in nonsyndromic CL/P but the specific nature of this relationship remains unclear. The results on the relationship between dermatoglyphic patterns and nonsyndromic cleft lip and palate have been fairly consistent. However, cleft lip and palate individuals seem to be characterized by an increased frequency of arches and ulnar loops and a decreased frequency of whorls. Orbicularis oris muscle defects are informative subclinical indicators of increased susceptibility to CL/P because preliminary studies have shown that the orbicularis oris muscle is hypoplastic in first-degree relatives of CL/P individuals than in controls. Many dental anomalies are associated with CL/P, ranging from a single malformed tooth in the vicinity of the cleft to dentition-wide reductions in tooth size or multiple congenitally missing teeth in both arches.

Multiple environmental factors are known to trigger and/ or modulate the cleft formation. The genetic components of cleft that underlie the susceptibility to respond to the environment still remain largely indefinite. Because of myriad of mendalian patterns exhibiting varying levels of penetrance, sex differences and environmental overlays, gene identification is difficult. [2] However, the current understanding of its complex nature supports the concept that nonsyndromic clefts are attributed to multiple interacting genes, with their own variable tendency to be expressed.[3–5] In addition, these disorders require the presence of appropriate environmental triggers for their expression. Although both physiologic/pharmacologic and genetic studies in animal models and human populations have identified several candidate genes and pathways that regulate transcription factors, growth factors, cell signalling and detoxification metabolisms,[6,7] the significance to nonsyndromic clefts of genetic variations such as single nucleotide polymorphisms (SNPs) in these genes is still unconfirmed.

Several genome-wide scans for nonsyndromic clefts and their associated or related traits have been carried out.[8–10] Most of these studies lack sufficient statistical power, and also they are not consistent in reporting genetic linkage to the same chromosomal region. The genes responsible for one form of X-linked (*CPX* [MIM 303400]) and one form of autosomal dominant (*CPI* [MIM 119530]) CL/P have been mapped to Chromosomes Xq13-q21.31[11,12] and 2q32[13] respectively. Pathogenic mutations were identified in the *TBX22* gene on Xq21.1. Genomic regions with evidence of linkage for nonsyndromic orofacial cleft (NSOFC) were identified at 6p24-p23 (*OFC1* [MIM 119530]),[14–16] 2p13 (*OFC2* [MIM 602966]),[8,17,18] 19q13 (*OFC3* [MIM 600757]),[18,19] 4q21-q31 (*OFC4* [MIM 608371]),[8,20] 4q16 (*OFC5* [MIM 608874]),[21–23] 1q32.3-q41 (*OFC6* [MIM 608864]),[24,25] 11q23.3 (*OFC7* [MIM 600644]),[26,27] 3q28 (OFC8 (603273)),[28,29] 13q33.1-q34 (OFC9 (610361))[9] and 2q33 (OFC10 (601912)),[30] but not all responsible genes are yet identified. Chromosomal aberrations involving Chromosomes 13 and 18 were reported to cause an increased incidence of clefts.[31]

Furthermore, disease-contributing alleles may also be present in genes for the *MSX2, RARA, TGFA, TGFB3, TGFB2, MTHFR, GABRB3, FOXE1, GLI2, JAG2, LHX8, PHF8, SATB2, SKI, SPRY2,* and *TBX10.*[22,32] However, none of these seem to play a major role in nonsyndromic CL/P, and they appear to be responsible for only a fraction of CL/P cases. [22] Most of these chromosomal regions contain more than one susceptibility locus for nonsyndromic clefts. Other linkage regions do not contain palpable candidate genes.

We performed a MEDLINE literature search by means of specific search terms (‘cleft lip’, ‘cleft palate’, ‘candidate genes’, ‘single nucleotide polymorphisms’, ‘aetiology’, and ‘genetics’) to identify relevant articles, primarily focusing on studies published till October 2008. The results summarize the latest information regarding the role of genetic influences in the development of nonsyndromic clefts and reflect our current information on some of the best-known genes that potentially contribute to the formation of clefts.

## LINKAGE STUDIES

Linkage analysis tests for cosegregation of a gene marker and disease phenotype within a family, to determine if the marker and the disease gene are physically linked. An array of family configurations can be tested, such as multiplex extended pedigrees and nuclear families with affected sib-pairs. Linkage studies have revealed at least 20 contributing chromosomal regions.[33] Scapoli *et al.*[34] found significant linkage disequilibrium between the GABRB3 gene and CL/P. Evidence for linkage to Chromosome 1p near the 5,10-methylenetetrahydrofoalte reductase locus and in the 1q21 and 1q32-42.3 regions has been reported for CL/P.[35] Regions on Chromosomes 6p, 2p, 4q and 17q have all shown some evidence of linkage to CL/P.[36] Beiraghi *et al.*[37] found linkage to 4q in one family and Mitchell *et al.*[38] showed evidence of association to this same region, but a later study showed evidence against linkage in this region.[39] Pezzetti *et al.*[40] reported a possible interaction between two regions that mapped in 6p23 and 2p13 in 38 CL/P multiplex families from Italy. However, Wong *et al*.[41] studied Swedish multiplex CL/P families, and found no evidence of linkage to selected candidate genes on Chromosomes 2, 4, 6 or 19. Similarly, Marazita *et al*.[8] did not find significant linkage in any of these regions in 36 CL/P multiplex Chinese families. Stein *et al*.[42] found significant linkage with BCL3 on Chromosome 19q in a fraction of their families. Wyszynski *et al.*[18] and Martinelli *et al.*[43] failed to find further evidence of linkage to this marker, but they did find a significant association for an allele at this marker using the transmission disequilibrium test (TDT).

## GENOME-WIDE SCREENS

Prescott *et al.*[15] first reported linkage of a genome-wide significance with two-stage genome-wide scan, using 92 UK sib pairs of CL/P, identified 11 loci on eight chromosomes using the 10-cM mapping in the first stage and seven of these loci were confirmed by a 5-cM mapping panel in the second stage. These eight chromosomal regions were on chromosomes: 1p (NPL = 2.35, *P* = 0.009, MLS = 1.51), 2p (NPL = 1.77, *P* = 0.04, MLS = 0.66), 6p (NPL = 2.35, *P* = 0.009, MLS = 1.34), 8q (NPL = 2.15, *P* = 0.015, MLS = 1.51) 11cen (NPL = 2.70, *P* = 0.003, MLS = 2.10), 12q (NPL = 2.08, *P* = 0.02, MLS = 1.5), 16p (NPL = 2.1, *P* = 0.018, MLS = 0.97) and Xcen-q (NPL = 2.40, *P* = 0.008, MLS = 2.68). Although none reached the level required for significant susceptibility loci, two of these areas have previously been implicated in CL/P, viz. 2p13, an area harbouring the TGFA gene, and 6p23-24. However, the statistical methods applied and the calculated *P* values have been criticized for not being stringent enough to support a genome-wide level of statistical significance. This is not surprising in view of the low genetic power expected from the study of 92 affected sibling pairs. The genome scan on Chinese multiplex families revealed positive multipoint results for regions on Chromosomes 1, 2, 3, 4, 6, 18 and 21 with the most significant results (HLOD = 2.0; α = 0.37) at 3q26 and 4q21. [8] Genome-wide scan of two large Syrian families detected a significant LOD score of 3.0 at 17p13.1.[44] Genome-wide scan on 10 multiplex families of Mexico, Argentina, and the United States yielded suggestive evidence of linkage to Chromosomes 2, 6, 17 and 18. Subsequent Fine mapping analysis showed intriguing evidence of linkage to 2q.[45] Field *et al.*[46] reported statistically significant two-point linkage results with markers on Chromosome 7 (LOD = 1.89), Chromosome 5 (LOD = 1.76), Chromosome 15 (LOD = 1.55), and Chromosome 20 (LOD = 1.46) in a genome-wide scan on 38 CL/P multiplex families of West Bengal. The linkage genome scan among 18 consanguineous CL/P Turkish families yielded statistically significant multipoint results from and regions on Chromosomes 4, 10, 12, and 15 with maximum multipoint HLOD's of 1.25, 1.30, 2.73, and 1.28 respectively.[47] Given that each individual study lacks statistical power for confirmatory linkages, various attempts have been made, using the method of meta-analysis, to combine the evidence from several studies on multiple markers in a particular candidate region and to summarize the multipoint LOD values evaluated at the location of each marker. However, this is not always practical because of the variability in methods and models between different studies. Marazita *et al.*[48] presented a meta-analysis of 13 10-cM genome scans of 388 extended multiplex families with CL/P from seven diverse populations (genotyped 2,551 individuals). The combination of evidence over multiple samples from 388 families, pinpointed highly significant loci contributing to nonsyndromic cleft lip and palate susceptibility with a maximum heterogeneity LOD score 6.6 near 9q21 and another (2q32-35) had a meta-analysis *P* value of 0.0004. A recent nonparametric linkage (NPL) analysis of two Indian pedigrees identified 11 genomic regions that could potentially harbour CL/P susceptibility variations with most significant evidence of linkage for Chromosome 13q33.1-34 at marker rs1830756 with heterogeneity LOD score of 5.57[9] and significant evidence of linkage for marker rs728683 on Chromosome 18q21.1 with LOD of 3.97 was identified that most likely harbours a high-risk variant for CL/P.[10] To determine statistical significance in genome-wide scans many laboratories use LOD > 3.7 and *P* < 2 × 10-5.[49] It is noteworthy that few of the genome-wide scans performed to date on nonsyndromic clefts could not meet the Lander and Kruglyak criteria[49] because of insufficient information content of the mapped locus (*i.e.* marker interval more than 1.5 cM). The small deletions or duplications of chromosome material can lead to orofacial clefts. Array-CGH has been developed as a method to identify and map sub-microscopic deletions/duplications simultaneously onto the genome sequence. The use of multiple probes simultaneously is now possible using probes of known location on the genome 1 Mb apart. Recent studies using array comparative genomic hybridization helped in identifying the novel candidate genes associated with cleft lip and palate.[50,51]

## ASSOCIATION STUDIES USING SINGLE NUCLEOTIDE POLYMORPHISMS

Association studies determine whether alleles occur together with a specific phenotype more often than in a control group. This was done most commonly by associating nonsyndromic cleft lip or palate to microsatellite markers or SNPs within specific transcription factors, growth factors, cell signalling and detoxification metabolisms regulating genes. A specific allele has to be suspected as disease-causing or protective and therefore tested in either a case-control study or a family-based association design. Clues towards candidate genes typically originate from basic biological studies such as animal models. Association studies provide a powerful tool for identifying alleles of minor importance. In this regard, using the case-control design, Ardinger *et al.*[17] found an increased risk for CL/P among cases carrying the *Taq1* C2 allele of the TGFA gene (OR = 3.18, 95% CI = 1.26 to 8.22). These results were later confirmed by two other small case-control studies: Holder *et al*. [52] and Sassani *et al*.[53] found an association between CL/P and the *Taq1* C2 allele (Chi-square = 15.04, 1 *df*, *P* = 0.001 and Chi-square = 5.08, *P* = 0.02, respectively). Mitchell[54] performed a meta-analysis utilizing results from all available studies throughout the year 1997 and found a statistically significant overall association between CL/P and TGFA genotype (OR 5 1.43, 95% CI 5 1.12 to 1.80). Romitti *et al*.[55] did not find evidence that the C2 allele at TGFA increased risk for either CL/P (OR 5 0.9, 95% CI 5 0.5 to 1.5) or CP (OR 5 1.0, 95% CI 5 0.4 to 2.0) in a case-control study from Iowa. A false association may be detected if the tested variant is not the actual disease-causing variant, but closely located to the disease-causing variant. False associations may also occur if the case and control groups show differences in the studied allele frequencies, not causally related to the studied disease, i.e. population stratification.[56] Because of the risk of population stratification, family-based association designs have become increasingly popular. A key susceptibility locus for nonsyndromic cleft lip with or without cleft palate on chromosome 8q24 was identified in a genome wide association study.[57] Case-control association studies have provided evidence that variants in the genes *MTHFR, ARNT, TGFA, GAD1, MSX1, RARA, TGFB3* are associated with the CL/P phenotype. Several studies using candidate genes involved in pathways that regulate transcription factors, growth factors, cell signalling and detoxification metabolisms are represented in Table 1.

**Table 1:** Studies using candidate genes involved in different pathways

*Gene name*	*Gene symbol*	*Chromosomal location*	*References*
Transcription factors			
Muscle segment homeobox homolog 1	MSX1	4p16.3-p16.1	21-23,32,49,123,124
Muscle segment homeobox homolog 2	MSX2	5q34-q35	22
T-box transcription factor TBX22	TBX22	Xq21.1	76,125
Interferon regulatory factor 6	IRF6	1q32.3-q41	24
T-box transcription factor TBX10	TBX10	11q13.2	22,125
Distal-less homeobox 2	DLX2	2q32	126
Distal-less homeobox 3	DLX3	17q21	125
SATB homeobox 2	SATB2	2q33	22,58,127
RYK receptor-like tyrosine kinase	RYK	3q22	128
Transcription factor AP-2 alpha	TFAP2A	6p24	129
Growth factor			
Transforming growth factor, alpha	TGFα	2p13	17,21,130-132
Transforming growth factor, beta 1	TGFβ1	19q13.1	132,133
Transforming growth factor, beta 3	TGFβ3	14q24	55,125,126,132,134
V-ski sarcoma viral oncogene homolog	SKI	1q22-q24	22,135
Vibroblast growth factor receptor 1	FGFR1	8p11.2-p11.1	61,136
Tumour protein p63	TP63	3q28	29,137
Cell signalling			
Poliovirus receptor-related 1	PVRL1	11q23.3	71,73,125,131,138
Poliovirus receptor-related 2	PVRL2	19q13.2	19
Poliovirus receptor	PVR	19q13.2	19
Patched homolog 1 (Drosophila)	PTCH	9q22.3	139
Gamma-aminobutyric acid a receptor, beta 3	GABRB3	15q11.2-q12	34,126,132
Aryl-hydrocarbon receptor nuclear translocator 2	ARNT2	15q24	140
Receptor tyrosine kinase-like orphan receptor 2	ROR2	9q22	48
Folate pathway			
5,10-methylenetetrahydrofolate reductase	MTHFR	1 p36.3	119,121,141-143
Methylenetetrahydrofolate dehydrogenase 1	MTHFD1	14q24	119,144
5-methyltetrahydrofolate-homocysteine methyltransferase	MTR	1q43	119,145
5-methyltetrahydrofolate-homocysteine methyltransferase reductase	MTRR	5p15.3-p15.2	145
Reduced folate carrier	RFC1	21q22.3	119,146
Folate receptor 1	FOLR1	11q13.3-q14.1	72,147
Betaine-homocysteine methyltransferase	BHMT	5q13.1-q15	148
Betaine-homocysteine methyltransferase 2	BHMT2	5q13	149
Detoxification			
Cytochrome P450, family 1, subfamily A, polypeptide 1	CYP1A1	15q22-q24	150,151
N-acetyltransferase 1	NAT1	8p23.1-p21.3	7,152
N-acetyltransferase 2	NAT2	8p22	7,151
Glutathione S-transferase M1	GSTM1	1p13.3	4
Glutathione S-transferase theta 1	GSTT1	22q11.23	4,151
Glutathione S-transferase pi	GSTP1	11 q13	153
Retinoic acid receptor, alpha	RARA	17q21	6,154-156
Epoxide hydrolase 1, microsomal	EPHX1	1q42.1	153

Recent technical progress has made it possible to perform high-throughput sequencing of candidate genes in large patient cohorts. Not only genes identified in syndromic CL/P, but also candidates selected on the basis of animal studies, linkage and cytogenetic studies have now been sequenced.[22] This may increase the number of genes known to contribute to CL/P substantially, but we still have important work to do in the field of biological causality.

## CONTRIBUTION OF SYNDROMES ASSOCIATED WITH OFC

Clefts associated with other syndromes also had given sufficient hints to map the genes underlying the isolated forms.[58] More than 300 syndromes are known to have either cleft lip or palate as an allied feature (http://www.ncbi.nlm.nih.gov/OMIM). One of the most common autosomal dominant disorders associated with CLP is Van derWoude syndrome (VWS) (OMIM 119300), associated with highly characteristic pitting of lower lip mucosa and CLP.[59] The locus for VWS has previously been mapped to Chromosome 1 (1q32-q41).[60] Recently, mutations in the interferon regulatory factor 6 (IRF6) gene were reported to underlie VWS,[60,61] and, subsequently, variants in IRF6 were found to be significantly associated with nonsyndromic clefting as well.[24,62] A recent study identified a mysterious SNP rs642961, in a previously unexplored enhancer region of the IRF-6 gene causing about 18 percent of CP malformation. [63] The Wolf-Hirschhorn syndrome (WHS)*(OMIM 194190)*, caused by partial deletion of the short arm of Chromosome 4 (4p), is characterized by severe growth retardation and mental defect, microcephaly. The craniofacial abnormalities include microcephaly, maxillary hypoplasia, hypertelorism, high nasal bridge with a characteristic Greek warrior helmet appearance, oral clefts, and dental anomalies.[64] Fluorescence *in situ* hybridization (FISH) analysis of eight Finnish patients with WHS was done; five patients lacked one copy of *MSX1* with oligodontia, while the other three had two hybridization signals. One of these presented with the only case of cleft palate.[65] A heterozygous MSX1 nonsense mutation has been identified in a three-generation Dutch family exhibiting various combinations of CLP, CP and selective tooth agenesis.[66] In a follow-up study using 1000 unrelated individuals with CLP, complete sequencing of the gene showed that mutations in MSX1 alone could account for 2% of isolated CL/P.[32]

Autosomal dominant form of Kallmann syndrome (KAL2) (OMIM 147950), which is characterized by hypogonadism and anosmia, and also clefting in 5-10% of the cases is caused by mutation in the gene encoding fibroblast growth factor receptor-1.[67] FGFR1 (fibroblast growth factor receptor-1), encodes a transmembrane receptor tyrosine kinase that transduces signals from secreted FGFs.[68] Dode *et al*.[69] confirmed that loss-of-function mutations in FGFR1 account for approximately 20% of all cases of Kallmann syndrome. Recent analysis of 12 genes involved in the fibroblast growth factor signalling pathway in nonsyndromic cleft lip or palate families revealed seven mutations in which structural analysis predicted functional impairment in the FGFR1, FGFR2, FGFR3, and FGF8 genes and causing 3 to 5% of nonsyndromic cleft lip or palate.[70] Cleft lip/palate ectodermal dysplasia syndrome (CLPED) is characterised by cleft lip with or without cleft palate, hidrotic ectodermal dysplasia, syndactyly, and occasionally mental retardation. The inheritance of CLPED appears to be autosomal recessive. Other syndromes such as Zlotogora-Ogur syndrome and Margarita Island ectodermal dysplasia are also classified as CLPED. Using positional cloning, Suzuki *et al*.[26] identified mutations of the poliovirus receptor like-1 gene (*PVRL1*) in CLPED families from Margarita Island, Israel, and Brazil. A non-sense mutation of *PVRL1 gene (*W185X) is a susceptible allele in clefting in Northern Venezuela and was not found in any of the nonsyndromic cleft populations.[71] Later, a study using 146 CLP patients and 100 unaffected individuals of Italian origin did not find the W185X mutation in any samples.[72] A common glycine allele of the G361V polymorphism was significantly over-transmitted among all orofacial clefting phenotypes in all Iowan, Danish, and Filipino families.[73] Many other syndromes which have the cleft as one of the allied abnormalities have given several hints to identify the genes such as TP63 (OMIM 603273; 3q27),[74] TBX22 (OMIM 300307; Xq12-q21),[75,76] FOXC2 (OMIM 602402; 16q24.3),[77,78] FOXE1(OMIM 602617; 9q22).[79]

## ANIMAL MODELS

The mouse model is the preferred mammalian animal model, because it has a short reproduction cycle and a known genomic sequence, very close to the human genomic sequence.[80] Many genetically manipulated mouse models such as knockout, knock-in, gene-trapped, spontaneous mutations and chemically induced mutations provided an excellent opportunity to study CL/P. Many CL/P mouse models have counterparts in syndromic CL/P forms found in humans. Animal models with clefts arising spontaneously or as a result of mutagenesis experiments provide another exciting avenue for gene mapping.[81] Indeed, studies in these animal models have helped to identify a series of genes essential for palatal formation. Endothelin-1 (EDN1) is synthesized by vascular endothelial cells and is found in plasma. EDN1 is released from an inactive transitional form in a step catalyzed by endothelin-converting enzyme (ECE). EDN1 knockout mice have shown craniofacial abnormalities, including cleft palate. [82] The mouse deficient in ECA or in endothelin-A receptor genes also has shown almost identical abnormalities to those of EDN1-deficient mice.[83] Whereas in zebra fish, EDN1 is expressed ventrally in the primordia of the pharyngeal arches and helps in patterning of pharyngeal cartilage development. By injecting the morpholino, Kimmel *et al*.[84] have demonstrated the role of EDN1 in the sizing of pharyngeal skeletal elements of the jaw and opercular regions in zebra fish. But genes involved in the endothelin pathway (EDN1, ECE1, EDNRA, EDNRB) have not shown linkage with orofacial clefts.[85] γ-Aminobutyric acid (GABA) is a major inhibitory neurotransmitter in the mammalian central nervous system. Mice homozygous for mutations in the GABA synthesizing enzyme Gad67 indicate a role for GABA function in the development of the palate.[86,87] Similarly, mice lacking TGF-beta 3 exhibit an incompletely penetrant failure of the palatal shelves to fuse, leading to cleft palate.[88] The Msx1 homeobox gene is expressed at diverse sites of epithelial-mesenchymal interaction during vertebrate embryogenesis, and has been implicated in signalling processes between tissue layers. Mice lacking Msx1 function manifest a cleft secondary palate, a deficiency of alveolar mandible and maxilla and a failure of tooth development.[89,90]

## GENETIC PREDISPOSITION TO ENVIRONMENTAL FACTORS

Since the first report on the possible involvement of the environmental component in clefting,[91] extensive screening for teratogens that cause clefts has been conducted. A genetic predisposition has long been observed. A foetus with an affected parent or sibling has a 2-4% risk for a development of oral cleft compared to the 0.15% risk for the general population. Anti-epileptic medications taken in pregnancy have been shown to be associated with oral clefts; and other environmental factors are suspected, including maternal smoking, heavy alcohol intake, infections, folic acid deficiency, and Vitamin A toxicity.[92,93]

## STRESS

Previous studies revealed increased risk of clefts among infants born to women with higher stress.[94,95] It has been hypothesized that maternal stressors may cause birth defects through increased production of corticosteroids. Animal studies in rodents and rabbits have demonstrated that high doses of corticosteroids consistently cause cleft palate.[93,94,96] Stressful life events have been shown to be associated with increased levels of maternal corticotrophin-releasing hormone and corticosteroid during pregnancy. [97,98] Increased risk of oral clefts was observed in the infants born to women who took corticosteroid medications during the first trimester of pregnancy.[99,100]

## SMOKING

Cleft lip and cleft palate is one of the congenital defects associated with maternal smoking. As early as 1979, Ericson *et al.*[101] found an association between smoking and oral clefts. Cigarette smoking during pregnancy was associated with cleft defects.[102,103] In contrast, some studies found no association with maternal smoking for any form of oral clefts. [55,104] Meta-analysis of 11 published studies revealed an overall odds ratio of 1.29 (95% CI= 1.18 to 1.42) for any oral cleft in children of women who smoked.[105] When maternal smoking was considered together with a certain genetic background, the combined effect was more significant. The maternal glutathione s-transferase θ-1 (*GSTT1*) genotype, combined with smoking, could significantly increase the risk of CLP with an odds ratio of 4.9.[106] Infant *MSX1* genotypes in combination with maternal smoking contributed to an elevated risk for CLP by 7.16 times.[107] Moreover, use of multivitamins also did not mitigate the risk of oral clefting associated with smoking.

## ALCOHOL

Seminal studies revealed the teratogenic effects of heavy alcohol exposure.[108] Abel[109] reported that 9-18% of infants with foetal alcohol syndrome possess cleft lip with or without cleft palate. Alcohol consumption during pregnancy was associated with cleft defects. [55,110] Low-level alcohol consumption, however, did not seem to increase the risk of orofacial clefts.[111] A recent population-based case-control study showed that even a low level of alcohol consumption during pregnancy was significantly associated with an elevated risk of orofacial clefts in the offspring.[112] The child carrying mutant ADH1C allele seems to have a protective effect against the risk of oral clefts, but the maternal genotype plays a less important role than the child's genotype.[113] CYP2E1 genotypes of mother and child showed reduced risks of nonsyndromic oral cleft in children carrying the CYP2E c1/c2 mutated allele.[114]

## FOLIC ACID

Maternal nutrition may act as a forecast for the foetus of the nutritional environment it will encounter after birth. Maternal nutrition during pregnancy also appears to play an important role in palate development. Palate development delay was observed in folic acid-deficient mice.[115] Low dietary intake of B-complex vitamins and exposure to deficient or excessive amounts of vitamin A, have been linked to increased risks of clefts. Methylenetetrahydrofolate reductase (MTFHR) catalyzes the conversion of 5, 10-methylenetetrahydrofolate to 5-methyltetrahydrofolate, which is a co-substrate for remethylation of homocysteine to methionine. Methylenetetrahydrofolate reductase deficiency leads to homocystinuria. Significantly higher serum homocysteine levels were detected in mothers of babies with OFC compared with mothers of unaffected babies.[116,117] It has also been hypothesized that genetic variants in the enzymes controlling folate metabolism might play a role in the susceptibility of oral clefts. The gene encoding the MTHFR enzyme is known to have at least two functional polymorphisms: *C677T* and *A1298C*. The C677T variant of the gene encoding the 5,10-methylenetetrahydrofolate reductase (MTHFR) enzyme affects folate status. Compared with homozygotes (CC) for the common MTHFR C677T variant, heterozygotes (CT) have been reported as having 65% of enzyme activity levels *in vitro* and 30% of homozygous variants (TT).[118] The results of investigations of the presence of the MTHFR C677T variant in the mother and CLP have been inconsistent. The results of both the case-control comparison and the family-based transmission disequilibrium test analysis revealed no association of the *MTHFR 677TT* genotype or the *677T* allele with CLP. [3,119,120] A few studies have shown statistically significant positive associations between 677TT genotype and OFCs.[121] Wilcox *et al.*[122] reported that at least 400 *μ*g/day of folic acid supplementation during early pregnancy was significantly associated with reduced risk of cleft lip even after adjustment for multivitamins, smoking, and other potential confounding factors.

## CONCLUSIONS

There is strong evidence that nonsyndromic orofacial clefts have genetic predisposition, but in the absence of a classic mendelian inheritance pattern. The orofacial clefts are caused by the interactions of multiple interacting genes, each gene having its own variable tendency to be expressed. In addition, the expression of orofacial clefts requires the presence of appropriate environmental triggers. Markers in more than 16 chromosomal regions have shown evidence of linkage to orofacial clefts or its related phenotypes in many genome-wide studies, suggesting that these genetic loci may contain major genes influencing the expression of orofacial clefts. These include, but are not exclusive to or limited to, genes that regulate transcription factors, growth factors, cell signalling and detoxification metabolisms. However, it remains to be determined whether polymorphisms in these genes account for the reported linkages in these regions. Studies are under way in many laboratories around the world to identify the disease-causing variations in these genes that account for the linkages discussed above. Although no major gene has been confirmed, it is noteworthy that the field of cleft palate genetics has evolved in a relatively short time into a research field. If successful, the genetic approach is likely to provide a new plane of understanding on the pathophysiology of the orofacial clefts. It is, however, clear that there is no single major genetic risk factor for the development of orofacial clefts; and the development of the orofacial clefts in an individual will depend on the interaction of several genes of moderate effect with environmental factors. Identifying specific genetic polymorphisms that influence orofacial cleft phenotypes will shed light on the molecular pathways involved in these complex disorders and provide a better understanding of the pathophysiology of orofacial clefts.

## GLOSSARY

### Allele:

An alternative form of a gene (a viable DNA) that is located at a specific position on a specific chromosome. Usually alleles are sequences that code for a gene, but sometimes the term is used to refer DNA sequence variants in non-functional or junk DNA. In a diploid organism, one that has two copies of each chromosome, two alleles make up the individual's genotype. An individual's genotype for that gene is the set of alleles it happens to possess. Different alleles and their combinations may result in different phenotypes.

### Association studies:

Genetic association studies assess correlations between genetic variants and disease phenotypes on a population scale, relying on linkage disequilibrium between genotyped markers and unknown disease loci.

### Candidate gene:

Any gene that is suspected of being involved in a disease. The gene may be a candidate because it is located in a particular chromosome region suspected of being involved in the disease or its protein product may suggest that it could be the disease gene in question. A candidate gene can also be identified by association with the phenotype and by linkage analysis to a region of the genome.

### Chromosome:

The DNA molecule is packaged into thread-like structures called chromosomes. Each chromosome is made up of DNA tightly coiled many times around proteins called histones that support its structure. Chromosomes in diploid organisms such as humans come in pairs; each member of a pair is inherited from one of the parents. Humans carry 23 pairs of chromosomes and are not visible even under microscope. Chromosomes can be distinguished by their length and by their banding pattern when stained with appropriate methods. Each chromosome has a constriction point called the centromere, which divides the chromosome into two sections, or “arms.” The short arm of the chromosome is labeled the “p arm.” The long arm of the chromosome is labeled the “q arm.” The location of the centromere on each chromosome gives the chromosome its characteristic shape, and can be used to help describe the location of specific genes.

### Codon:

A set of three adjacent nucleotides, also called triplet, in mRNA that base-pair with the corresponding aniticodon of tRNA molecule that carries a particular amino acid, hence, specifying the type and sequence of amino acids for protein synthesis. All 64 possible 3-letter combinations of the DNA coding units T, C, A and G are used either to encode one of these amino acids or as one of the three stop codons that signals the end of a sequence. While DNA can be decoded unambiguously, it is not possible to predict a DNA sequence from its protein sequence. Because most amino acids have multiple codons, a number of possible DNA sequences might represent the same protein sequence.

### Complex traits:

Complex traits are features whose properties are controlled by many genes. These are also known as polygenic traits. Polygenic inheritance, also known as quantitative or multi-factorial inheritance refers to inheritance of a phenotypic characteristic that is attributable to two or more genes and their interaction with the environment. Unlike monogenic traits, polygenic traits do not follow patterns of Mendelian inheritance.

### Epigenetic effect:

The term epigenetics is used to describe the study of heritable changes in genome function that occur without a change in DNA sequence. This includes; the study of how patterns of gene expression are passed from one cell to its descendants, how gene expression changes during the differentiation of one cell type into another, and how environmental factors can change the way genes are expressed. DNA methylation is one such change, which can turn off the expression of some genes.

### Familial aggregation:

Occurrence of a trait in more members of a family than can be readily accounted for by chance. However, disease also can aggregate in families because nongenetic risk factors cluster among relatives. A key measure of clustering or aggregation in families is the recurrence risk, that is, the risk of disease experienced by relatives of a subject with disease.

### Gene:

The functional and physical unit of heredity passed from parent to offspring. Genes are pieces of DNA, and most genes contain the information for making a specific protein. DNA segment is transcribed into messenger RNA and is finally translated into a protein. Genes comprise the exons that are actually translated (coding regions) and separated by long regions of DNA called introns.

### Genetic heterogeneity:

The production of the same or similar phenotypes by different genes or different genetic mechanisms in different pedigrees. If different alleles at a locus produce variable expression of a condition is known as allelic heterogeneity and if mutations at different loci produce the same disease phenotype is known as locus heterogeneity.

### Genetic polymorphism:

A variation in the DNA that is too common to be due merely to new mutation. A polymorphism must have a frequency of at least 1% in the population. The development of DNA markers allowed human gene mapping to start in earnest.

### Genome:

The genome of an organism is a complete genetic sequence on one set of chromosomes or one of the two sets that a diploid individual carries in every somatic cell. The term genome can also be applied to that stored within organelles that contain their own DNA, as with the mitochondrial genome.

### Genome-wide association:

A genome-wide association study is defined as any study of genetic variation across the entire human genome that is designed to identify genetic associations with observable traits, or the presence or absence of a disease or condition.

### Haplotype:

A set of closely linked allelic state present on one chromosome which tend to be inherited together. It refers to a collection of specific alleles in a cluster of tightly-linked genes. Haplotypes can be broken down by recombination. The tighter the linkage, the less likely that the haplotype will be broken up by recombination, and will be preserved through subsequent generations.

### Linkage analysis:

By using the linkage analysis we can trace the pattern of heredity in large, high-risk families, and to locate a disease causing gene mutation by identifying traits that are co-inherited with it. Parametric linkage analysis is usually used to find chromosomal regions linked to a disease that is described with a specific genetic model. This is done by investigating the relations between the disease and genetic markers, that is, well-characterized loci of known position with a clear Mendelian mode of inheritance. When we are studying complex traits such as nonsyndromic cleft lip and or palate we usually do not know the mode of inheritance. In this case we use non-parametric linkage analysis where we do not need to specify the penetrance model or disease allele frequency. Parametric analysis focuses on the co-segregation of the marker and the trait phenotype. Non-parametric analysis focuses on allele-sharing distribution between relatives that share the trait phenotype of interest.

### Linkage disequilibrium:

Linkage disequilibrium describes a situation in which some combinations of alleles or genetic markers occur more or less frequently in a population than would be expected from a random formation of haplotypes from alleles based on their frequencies. Non-random associations between polymorphisms at different loci are measured by the degree of linkage disequilibrium (LD). Alleles at markers near disease causing genes tend to be in linkage disequilibrium in the affected individuals.

### Linkage:

Linkage is a physical phenomenon that is due to the physical location of loci on a chromosome. Two loci are said to be linked if sufficiently close to each other on the same chromosome and alleles at these loci co-segregate more often than expected by independent assortment.

### LOD score:

LOD stands for logarithm of the odds (to the base 10). This is a measure of the likelihood of genetic linkage between loci. For a mendelian character, a lod score greater than +3 is evidence of linkage; one that is less than −2 is evidence against linkage.

### Microsatellites:

Microsatellites are polymorphic loci present in nuclear and organellar DNA that consist of repeating units of 1-6 base pairs in length. They are typically neutral, co-dominant and are used as molecular markers which have wide-ranging applications in the field of genetics, including kinship and population studies.

### Minisatellites:

A minisatellite is a section of DNA that consists of a short series of bases 10-60bp.These are also known as variable number tandem repeats (VNTRs). The VNTRs have many alleles and high heterozygosity. The VNTRs are not evenly spread across the genome and are not generally found in coding DNA.

### Mutation:

A mutation is a permanent change in the DNA sequence of a gene. Mutations in a gene's DNA sequence can alter the amino acid sequence of the protein encoded by the gene. Mutations occur more-or-less randomly throughout the genome and may affect any gene. In a clinical sense, any mutation that disrupts the information contained in DNA and leads to disease. The mechanisms leading to mutations are diverse: from exogen and endogen carcinogens to DNA repair defects. Mutation may be the insertion (addition) or deletion of one or a few nucleotides. An insertion is the integration of an additional fragment into the DNA, deletion is the elimination of a DNA fragment. Inversion is the reversal of a DNA fragment by excision and re-integration in the opposite direction. Substitution is the replacement of a base (A,T,C, or G) with another. A mutation can be a change in single base pairs or can involve a longer DNA fragment. The length of the changed DNA does not necessarily correspond with the impact of the mutation: in some cases, the exchange of a single base pair can have devastating consequences while, in other cases, a change in a long DNA segment may have no recognisable effect at all.

### Penetrance:

Penetrance is a term used in genetics describing the proportion of individuals carrying a particular variation of a gene (allele or genotype) that also expresses a particular phenotype. A penetrance of 100% means that the associated phenotype always occurs when the corresponding genotype is present. Similarly, if only 50% of those carrying a particular allele exhibit a phenotype, the penetrance is 50%.

### Recombination:

Recombination is an event, occurring by the crossing-over of chromosomes during meiosis, in which DNA is exchanged between a pair of chromosomes. Thus two genes that were previously unlinked, being on separate chromosomes, can become linked because of recombination; and vice versa: linked genes may become unlinked. This process leads to offspring having different combinations of genes from their parents and can produce new chimeric alleles.

### Restriction fragment length polymorphisms (RFLPs):

The first generation of DNA markers was RFLPs. RFLPs are based on the analysis of patters derived from a DNA sequence cleaved by using known restriction enzymes. Differences are noticed when the length of fragments are not the same, telling us that the restriction enzyme cut the DNA at two unrelated locations. RFLPs have only two alleles: the site is present or it is absent.

### Single nucleotide polymorphisms (SNPs):

SNP is a DNA sequence variation occurring when a single nucleotide - A, T, C, or G - in the genome differs between members of a species or between paired chromosomes of an individual. Single nucleotide polymorphisms may fall within coding sequences of genes, non-coding regions of genes, or in the intergenic regions between genes. SNPs within a coding sequence will not necessarily change the amino acid sequence of the protein that is produced, due to degeneracy of the genetic code.

### Whole genome scan:

Linkage analysis in which markers placed at regular intervals and covering the whole genome are typed. It is often the first approach when no genetic information is available about a particular phenotype.
